# Meta Analysis of Efficacy and Safety of Prostate Biopsy: A Comparison Between Transperineal and Transrectal Approach

**DOI:** 10.5152/tud.2025.24094

**Published:** 2025-01-03

**Authors:** Syah Mirsya Warli, Stivano Rizky Valentino Torry, Dhirajaya Dharma Kadar, Ginanda Putra Siregar, Fauriski Febrian Prapiska

**Affiliations:** 1 Division of Urology, Department of Surgery, Faculty of Medicine, Universitas Sumatera Utara – Haji Adam Malik General Hospital, Indonesia; 2 Department of Urology, Faculty of Medicine, Universitas Indonesia – Haji Adam Malik General Hospital, Medan, Indonesia; 3 Department of Urology, Universitas Sumatera Utara Hospital – Universitas Sumatera Utara, Indonesia

**Keywords:** Prostate biopsy, prostate cancer, transperineal, transrectal

## Abstract

Improved prostate biopsy procedures have been developed to overcome traditional limitations, aiming to enhance cancer diagnosis accuracy. To assess the existing knowledge of the effectiveness and risks linked to transperineal (TP) vs. transrectal (TR) prostate biopsy. Approaches: a comprehensive search was carried out in PubMed, Embase, Web of Science, and Cochrane Library to locate all pertinent papers published till June 8, 2023. Data on cancer detection rate and complications after prostate biopsy were gathered and analyzed via Review Manager software. A subgroup analysis was conducted to evaluate the influence of the study type. A total of 19 publications, involving 80 133 patients with suspicion of prostate cancer who underwent prostate biopsy, were enrolled in the analysis. The pooled estimate demonstrated no significant differences in the cancer detection rate between TR and TP (risk difference (RD) = 0.03; 95% CI: −0.01 to 0.08; *P *= .12). In terms of complications, the TP approach significantly decreased the risk of rectal bleeding (odds ratio (OR) = 0.24; 95% CI: 0.12-0.51; *P* < .001), fever and urinary tract infection (OR = 0.28; 95% CI: 0.15-0.52; *P *< .001), and sepsis (OR = 0.50; 95% CI: 0.28-0.90; *P* = .02) compared to the TR approach. In conclusion, there was no significant disparity in the cancer detection rate between TP and TR approaches. However, the TP strategy exhibited an advantage over TR due to a reduced risk of infection and rectal bleeding. Further research is needed to validate these results and develop a more efficient diagnostic approach for prostate cancer.

**Main Points**

- The study compares transperineal (TP) and transrectal (TR) prostate biopsies, finding no significant difference in cancer detection rates but highlighting the superior safety profile of the TP approach.
- TP prostate biopsy significantly reduces the risk of rectal bleeding, fever/urinary tract infections, and sepsis compared to the TR method.
  This meta-analysis includes data from 19 studies involving 80,133 patients, providing robust statistical evidence and subgroup analyses to validate results.
- Advanced statistical methods, including risk difference and odds ratio assessments, were employed, with a rigorous quality assessment of included studies using NOS and Jadad scoring systems.

## Introduction

Prostate cancer is the second most prevalent malignancy in males and the fifth leading cause of death worldwide.^1^ In 2021, there were approximately 248 530 cases of prostate cancer, representing more than 26% of new cancer diagnoses.^2^ Aside from digital rectal examination (DRE), diagnostic imaging methods, and prostate-specific antigen (PSA) testing, histopathology is regarded as the most dependable method for diagnosing prostate cancer. The systematic sextant prostate biopsy, guided by transrectal ultrasonography, is a widely accepted and often conducted method for detecting prostate cancer.^3^ Traditional prostate biopsies have disadvantages such as serious side effects and a significant rate of false-negative results. Technological progress has decreased the reliance on systematic sextant prostate biopsy for prostate cancer diagnosis. Using 12 cores, along with additional lateral cores, has demonstrated a greater cancer detection rate compared to the sextant biopsy. Having over 12 cores does not provide any substantial advantage.^4^ Prostate biopsy using magnetic resonance imaging (MRI)-ultrasound fusion is more effective than cognitive registration and traditional biopsy in detecting prostate cancer and is associated with identifying higher cancer grades.^5^ Advanced diagnostic techniques are critically needed to detect prostate cancer early due to its high rates of morbidity and mortality. The optimal biopsy method for identifying prostate cancer has not been conclusively established. The primary techniques used for prostate biopsy are transperineal (TP) and transrectal (TR) biopsy.^6^ The TR route is currently the most common technique in most countries, but TP biopsy is used increasingly, because TP biopsies are reported to have a lower risk of sepsis than TR biopsies.^7^ Transrectal prostate biopsy is associated with risks including rectal bleeding, fever, sepsis, hematuria, and acute urinary retention.^8,9^ The European Association of Urology recommends adopting TP prostate biopsy over TR prostate biopsy because of its reduced complication rate.^10^ Several studies have examined the cancer detection and complication rates of TP and TR techniques; however, the results are inconsistent.^11,12^ Some studies reported significantly higher cancer detection rates with TP, and some did not detect any statistically significant alterations in sensitivity.^13^ This systematic review and meta-analysis aimed to compare the effectiveness and risks of TR and TP approaches by analyzing all published randomized controlled trials (RCTs) and non-randomized controlled trials (non-RCTs) to offer more precise and convincing evidence on the best prostate biopsy method for detecting prostate cancer.

## Material and Methods

The systematic review was registered in the International Prospective Register of Systematic Reviews database with the registration number CRD42024553995. The study was carried out following the 2020 standards of the Preferred Reporting Items for Systematic Reviews and Meta-Analyses (PRISMA).^14^

### Database Search

Systematic searches were conducted in PubMed, Embase, Web of Science, and the Cochrane Central Register of Controlled Trials (CENTRAL) to identify relevant studies published between January 2003 and July 8, 2023, using the search terms: (prostate*) AND (biopsy) AND (“transrectal” OR “rectal”) AND (“transperineal” OR “perineal”). The search results were restricted to studies published in peer-reviewed journals, written in English, and focused on adult human participants aged 18 years and above. The pertinent articles were imported into Mendeley reference manager and underwent digital deduplication, followed by manual deduplication. The last group of articles was transferred to Microsoft Excel for assessing titles and abstracts before moving on to evaluate the entire contents.

### Study Selection

Two independent authors assessed the titles and abstracts to ensure they met the research eligibility criteria. Articles were required to fulfill specific qualifying criteria: adult patients, 18 years and older, who are suspected of having prostate cancer and have already had a prostate biopsy. The study investigates issues in TP and TR prostate biopsies using empirical research methods such as RCTs, non-RCTs, case–control studies, and cohort studies. The research must be published in a peer-reviewed journal and available in English. The primary exclusion criteria were as follows: (1) insufficient information or comprehensive data; (2) unoriginal investigation; (3) patients having a background of prostate cancer, acute prostatitis, or verified urinary tract infection; and (4) repetition of prior publications. Disagreements were settled through discourse.

### Data Extraction

The 2 co-authors systematically assessed the selected studies individually to see if each study matched the inclusion criteria. The data were gathered centrally from the appropriate articles. The collected data were standardized and consisted of the primary author’s name, publication year, research design, study location, patient numbers in each group, average age or age range, and core biopsy volumes. The main aim of the study was to examine the adverse effects and complications of prostate biopsy performed by TP or TR approaches. Disagreements during screening or data extraction were handled by author deliberation. If agreement was not achieved, a third impartial author acted as an arbiter to make the ultimate decision.

### Assessment of Risk of Bias

Two reviewers assessed the study quality using the Newcastle–Ottawa scale (NOS) for observational studies and the Jadad score for RCTs. The original NOS method assesses observational studies based on three criteria: sample selection, study comparability, and research outcome. The original NOS comprises 8 components with scores that vary from 0 to 9. The score ranges are as follows: 0-3 for low-quality investigations, 4-6 for moderate quality, and 7-9 for good quality.^15^ The Jadad score assesses the quality of RCTs using 3 criteria: dropouts and withdrawals (0-1 points), blinding (0-2 points), and randomization (0-2 points). Each component is assigned a score of either “yes” (1 point) or “no” (0 points). Superior scores correlate with improved reporting quality. The total score ranges from 0 to 5. Studies are considered to have “good” quality if they achieve a Jadad score of 3 or higher, and “poor” quality if they score 2 or lower.^16^ The authors came to a resolution to resolve their disputes. If consensus was not reached, the third author was responsible for resolving the issue.

### Statistical Analysis

The binary data from all relevant trials were displayed as risk difference (RD) or odds ratio (OR) with 95% CIs to compare the 2 techniques, TP and TR. Pooled RDs were used to assess the prostate cancer detection rate, while pooled ORs were used to analyze the problems and side effects associated with prostate biopsy when comparing TP and TR procedures. The Mantel–Haenszel-type approach was utilized to calculate the combined RD and OR for all subgroups. The meta-analysis assessed heterogeneity by the *I*-square test, which measures the percentage of variability in study outcomes attributed to heterogeneity rather than random chance. A random-effects meta-analysis was conducted to account for the variability in outcomes among the papers included, which differed in research design and location. It is advisable to conduct a sensitivity analysis, a technique used to assess result stability by excluding one study at a time, particularly when low-quality trials are included in the analysis. Publication bias was assessed by analyzing the symmetry of Begg’s funnel plots. The *P*-values were 2-sided and deemed statistically significant if they were below .05. The statistical data analysis was performed with Review Manager version 5.4, developed by The Cochrane Collaboration at The Nordic Cochrane Centre in Copenhagen, Denmark.

## Results

### Study Characteristics

**Figure 1.**
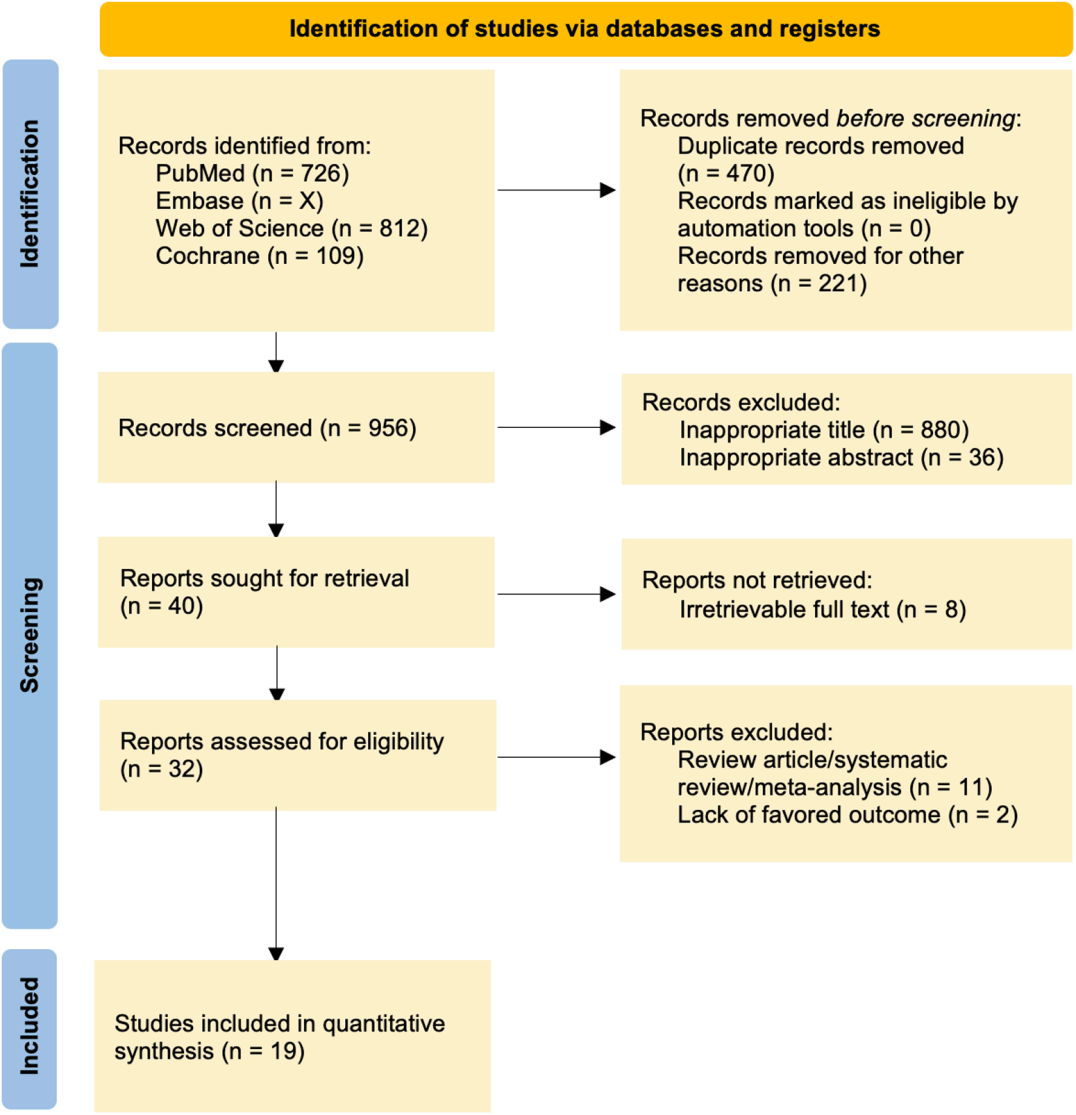
PRISMA flow chart of the study selection process.

Thousand six hundred forty-seven entries were identified in the database during the initial search for this investigation. After eliminating 470 duplicates, a total of 956 items were examined, leading to the removal of 880 titles and 36 abstracts. Eight reports were completely inaccessible and thus deemed irretrievable. We assessed 32 reports that satisfied the eligibility criteria. The systematic review’s screening process was successfully conducted, leading to the incorporation of 19 studies. The study consisted of 5 RCTs,^17-21^ 5 prospective observational studies,^22-26^ and 9 retrospective observational studies.^7,27-34^ Figure 1 displays the PRISMA flow diagram, presenting the details of the studies included in this analysis.

Table 1 outlines the attributes of 19 trials with 80 133 people who were suspected of having prostate cancer and underwent a prostate biopsy. About 16 664 individuals had a TP prostate biopsy, whereas 63 469 had a TR biopsy. The research was carried out on 5 continents: Asia (n = 10), Europe (n = 5), Australia (n = 2), North America (n = 1), and South America (n = 1). Fourteen observational studies were assessed using the NOS approach. Four studies were rated as having intermediate quality, and the other 10 were rated as having good quality. After evaluating 5 RCTs using the Jadad score, it was determined that 2 studies were of high quality; however, the other 3 RCTs were considered low quality due to deficiencies in randomization techniques and blinding. The quality assessment of each study was conducted using the NOS tool and Jadad score, and the results are presented in Tables 2 and 3, respectively.

### Prostate Cancer Detection Rate

**Figure 2.**
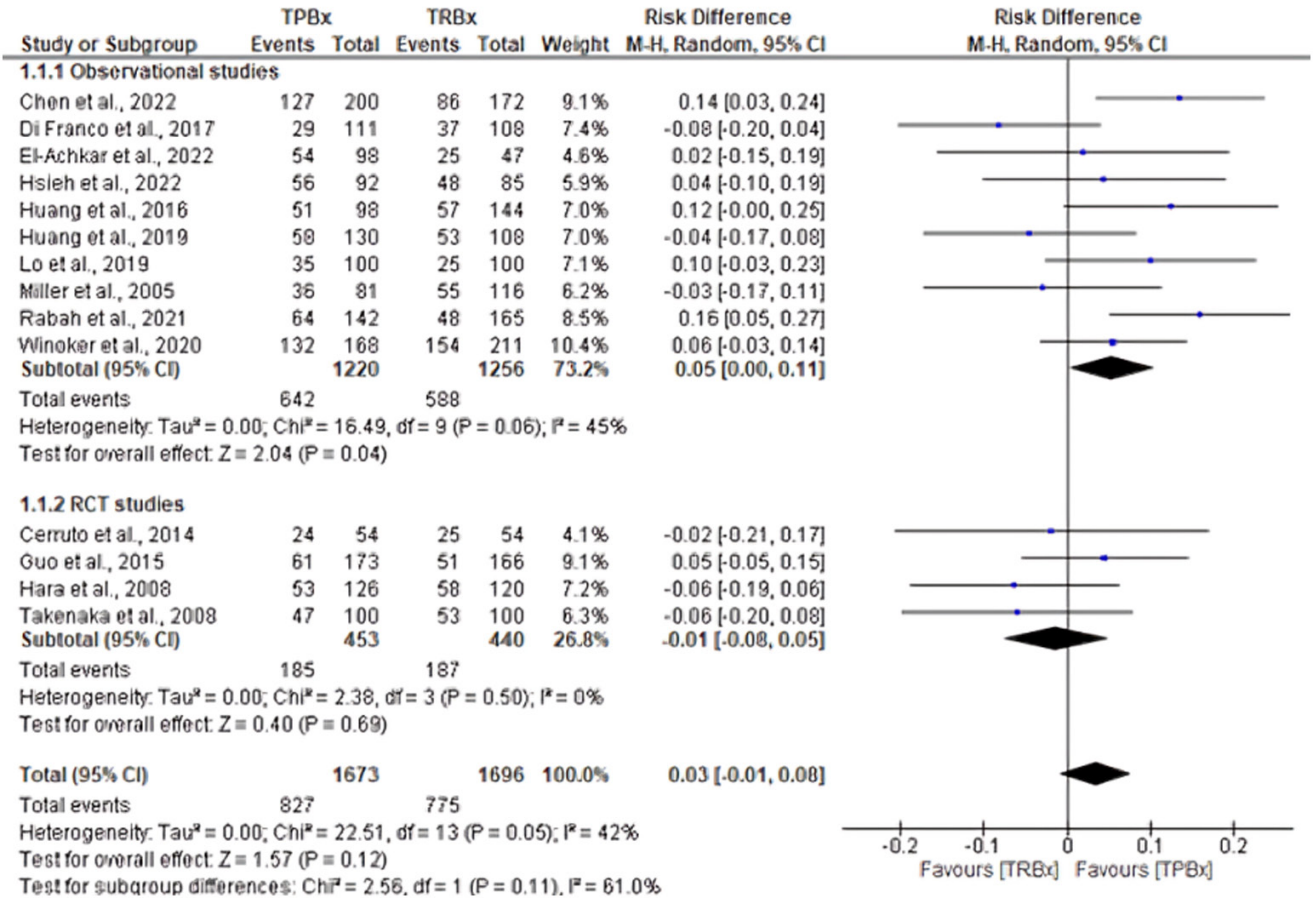
Forest plot comparing the detection rate of prostate cancer using transperineal and transrectal prostate biopsy. TPBx is short for transperineal biopsy, whereas TRBx is short for transrectal biopsy.

Data on cancer detection rates from 10 observational studies and 4 RCTs comparing TP vs. TR prostate biopsy were collected. Analyzed data from 14 trials with 3369 patients who had prostate biopsies showed no significant difference in cancer detection rates between TP (1673/3369) and TR (1696/3369) procedures (RD = 0.03; 95% CI: −0.01 to 0.08; *P *= .12). Subgroup analysis based on study design showed a consistent result in RCTs with a RD of −0.01 (95% CI: −0.08 to 0.05; *P *= .69). A notable difference was found in the subgroup analysis of observational studies, with a RD of 0.05 (95% CI: 0.00-0.11; *P* = .04) as depicted in Figure 2.

### Complications

**Figure 3.**
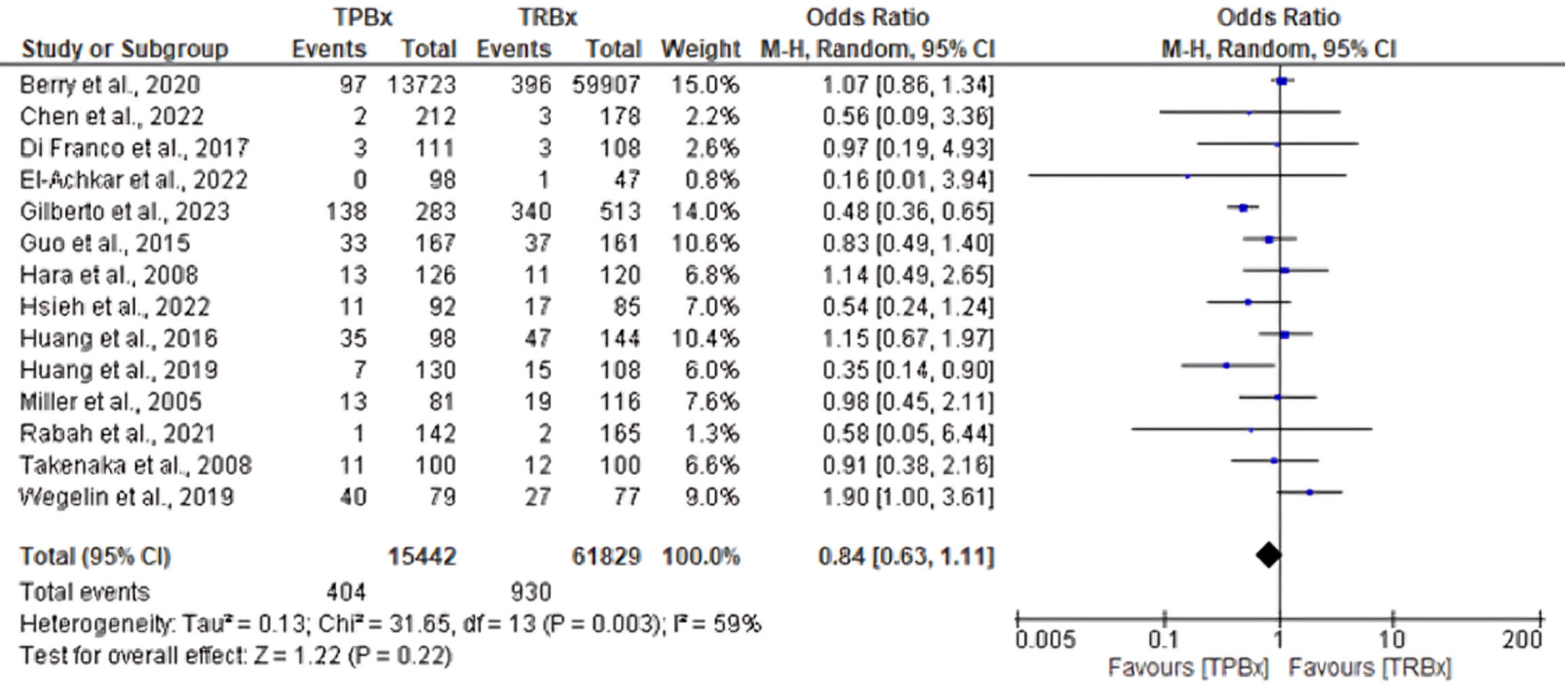
Forest plot comparing hematuria complications between transperineal and transrectal prostate biopsy. TPBx stands for transperineal biopsy while TRBx stands for transrectal biopsy.

**Figure 4.**
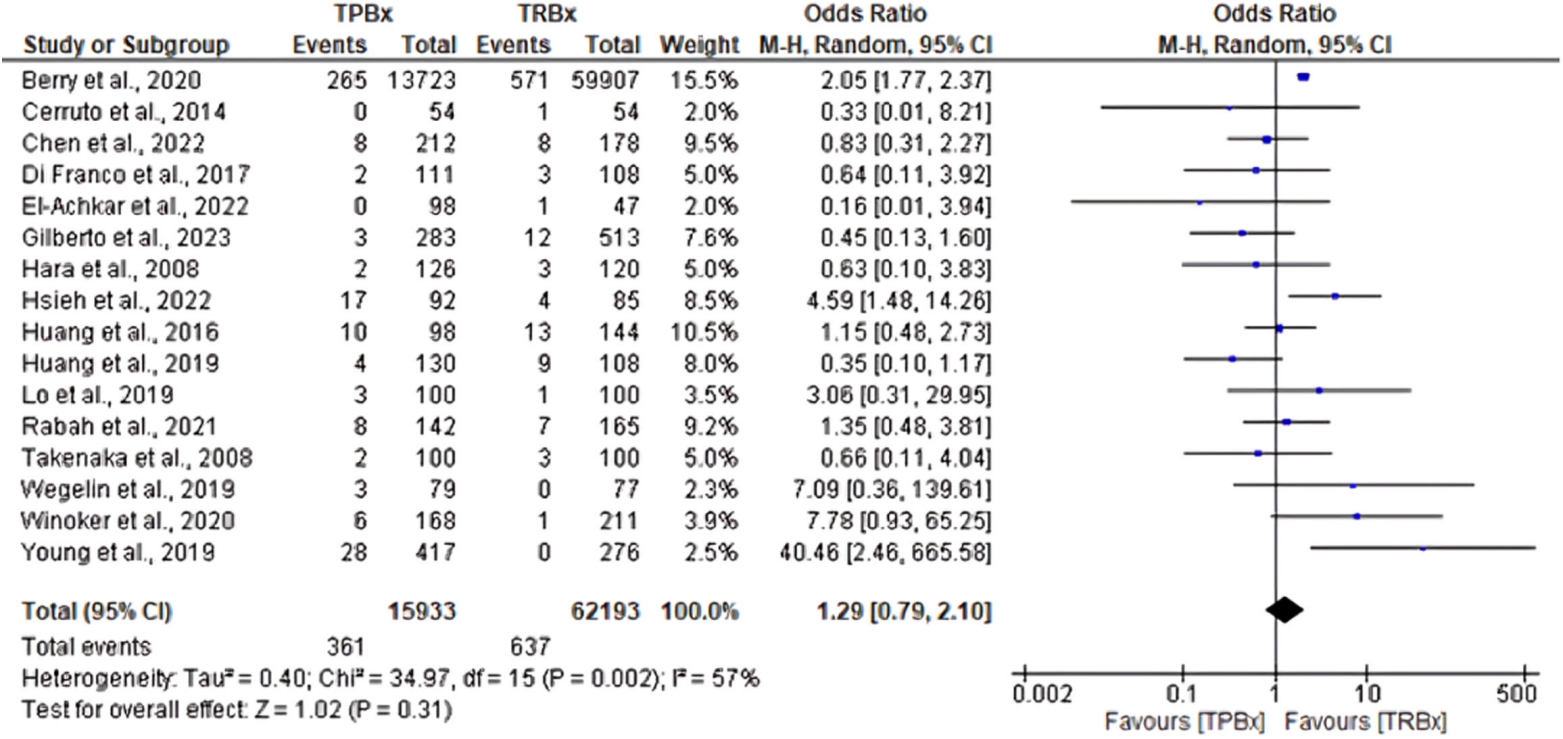
Forest plot comparing the incidence of acute urinary retention after transperineal and transrectal prostate biopsies. TPBx is short for transperineal biopsy, whereas TRBx is short for transrectal biopsy.

**Figure 5.**
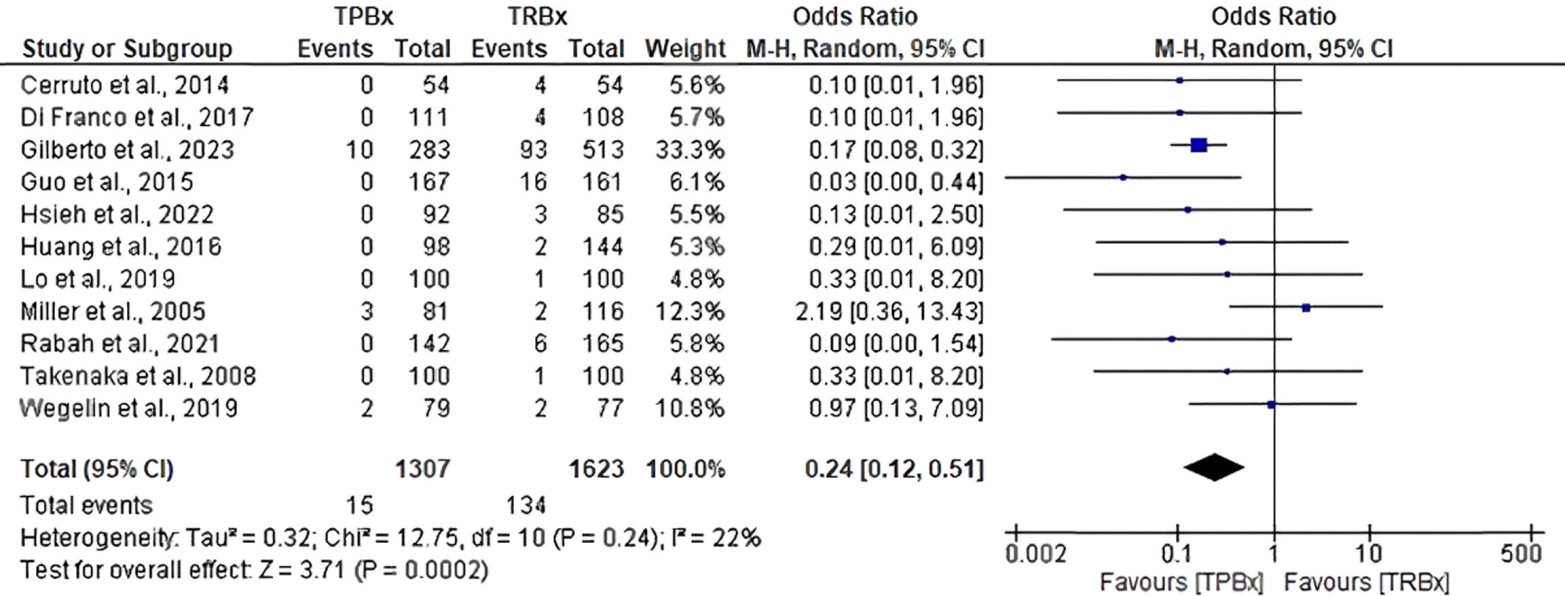
Forest plot comparing rectal bleeding complications between transperineal and transrectal prostate biopsies. TPBx stands for transperineal biopsy, while TRBx stands for transrectal biopsy.

**Figure 6.**
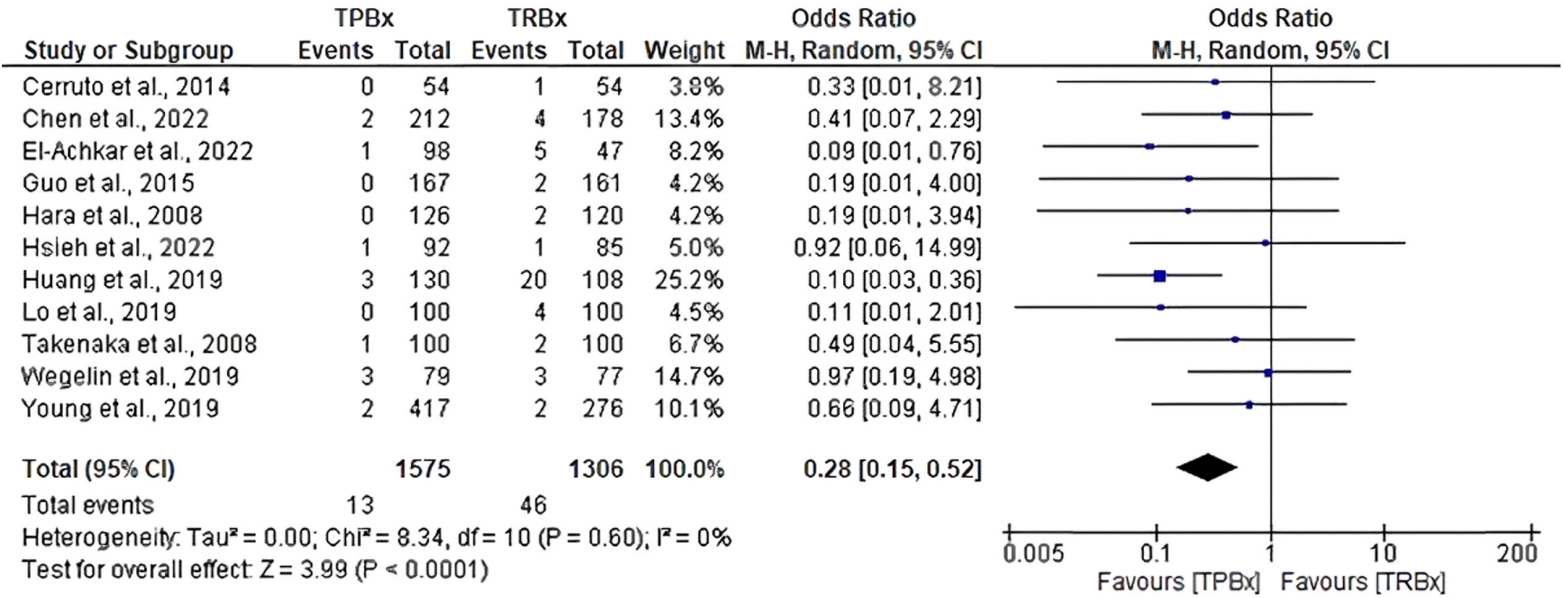
Forest plot comparing complication rates (fever-UTI) between transperineal and transrectal prostate biopsy. TPBx stands for transperineal biopsy, while TRBx stands for transrectal biopsy.

**Figure 7.**
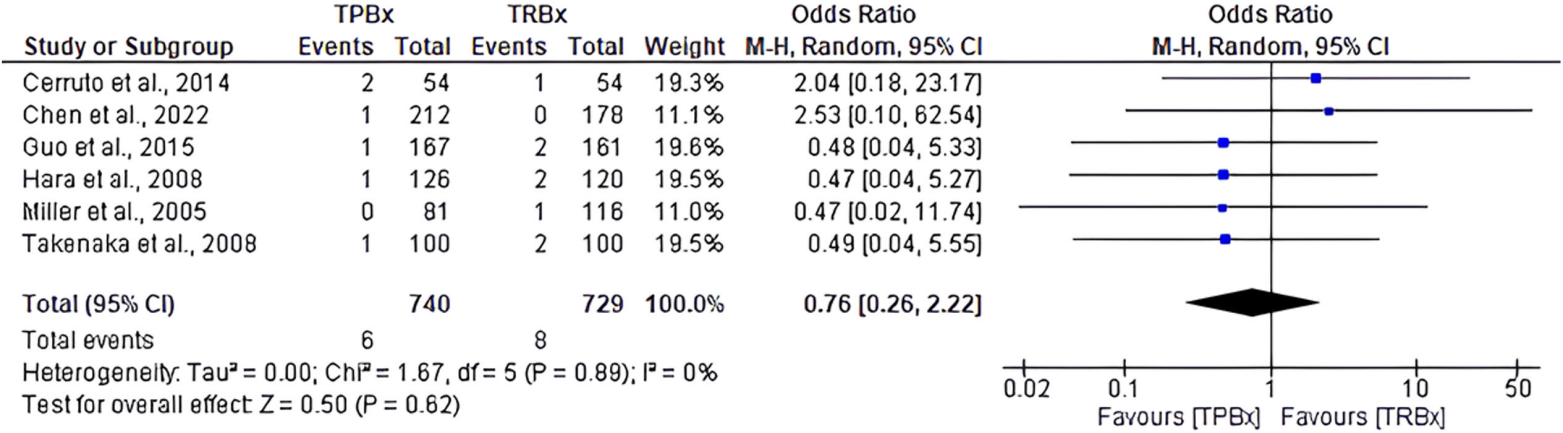
Forest plot comparing the occurrence of vasovagal events in transperineal and transrectal prostate biopsies. TPBx stands for transperineal biopsy while TRBx stands for transrectal biopsy.

**Figure 8.**
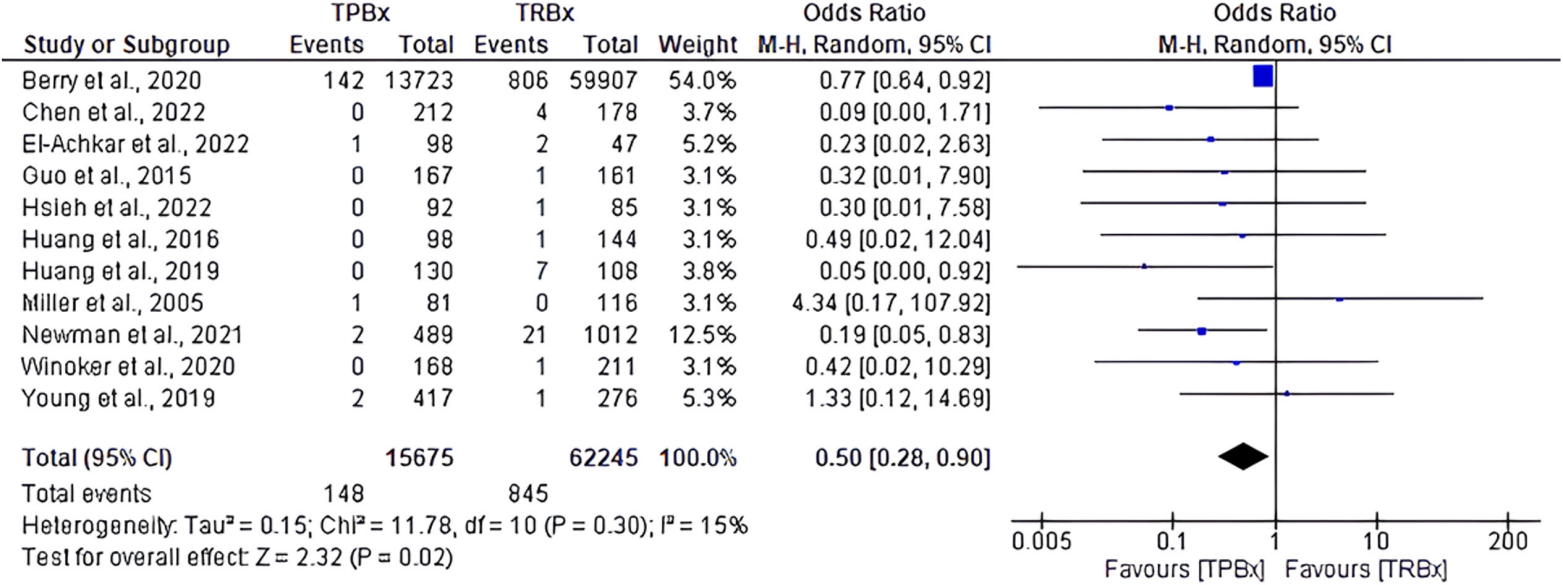
Forest plot comparing sepsis complication rates between transperineal and transrectal prostate biopsy. TPBx stands for transperineal biopsy, while TRBx stands for transrectal biopsy.

**Figure 9.**
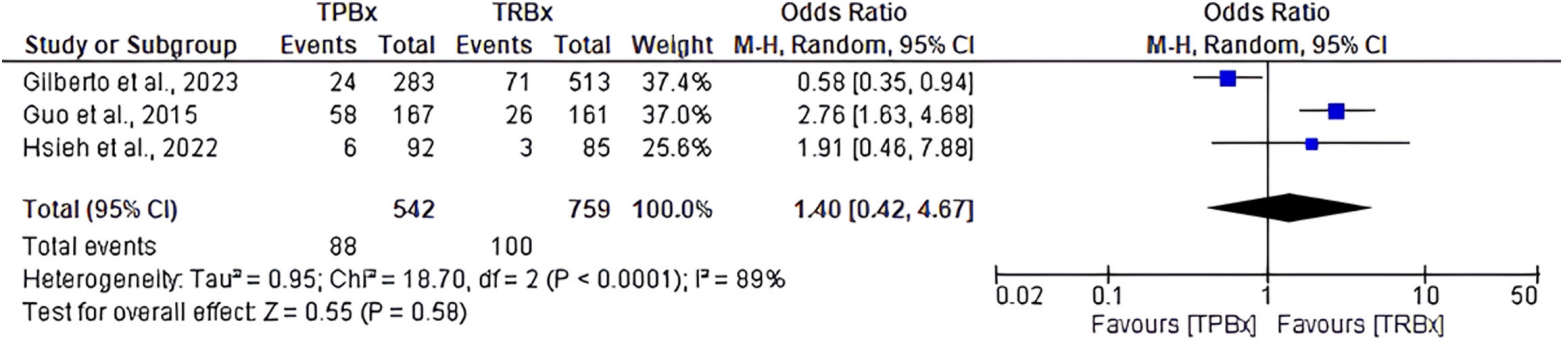
Forest plot comparing pain complications between transperineal and transrectal prostate biopsy. TPBx stands for transperineal biopsy, while TRBx stands for transrectal biopsy.

**Figure 10.**
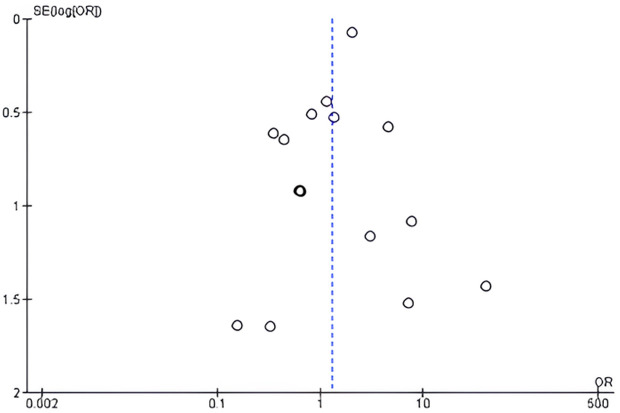
Funnel plot comparing outcomes of transperineal and transrectal prostate biopsies. TPBx stands for transperineal biopsy, while TRBx stands for transrectal biopsy.

All publications in the meta-analysis examined complications between TP and TR prostate biopsy groups. Aggregating multiple analyses of complications revealed that utilizing a TP approach for prostate biopsy significantly decreased the likelihood of rectal bleeding (OR = 0.24; 95% CI: 0.12-0.51; *P* < .001), fever and urinary tract infection (OR = 0.28; 95% CI: 0.15-0.52; *P* < .001), and sepsis (OR = 0.50; 95% CI: 0.28-0.90; *P* = .02) post-procedure. No notable disparities were noted in hematuria, acute urine retention, vasovagal event, and discomfort between the 2 therapies. The summary of complication outcomes and Clavien–Dindo classification^35^ are presented in Table 4. The comparison of each complication that occurs between TP and TR prostate biopsy is presented in the forest plot in Figures 3-9. The outcome comparison is depicted in the funnel plot in Figure 10.

## Discussion

Prostate biopsy is crucial for detecting prostate cancer and evaluating the grade and aggressiveness of the illness. Therapy plans for individuals are often based on biopsy results, clinical stage, and PSA levels.^36^ As a result, a prostate biopsy is essential for the diagnosis and treatment of prostate cancer.^37^ Two primary biopsy techniques, TP and TR, are used for diagnosing prostate cancer, each with different rates of detection and outcomes. This study aims to compare the effectiveness and side effects of TP and TR prostate biopsies through a systematic review and meta-analysis to address any discrepancies.

In accordance with the previous meta-analysis, it is indicated that there was no statistically significant disparity in the overall cancer detection rate between the TP and TR techniques (RD = 0.03; 95% CI: −0.01 to 0.08; *P* = .12).^12^ The meta-analysis of individual patient data found that TP prostate biopsy has a greater cancer detection rate in observational studies compared to the TR technique. However, this difference is not shown in RCTs, making the interpretation of these results challenging. Variations in prostate function across individuals may influence these results. During a TP biopsy, the core is guided longitudinally across the peripheral zone and anterior portion of the prostate, potentially increasing its sensitivity to prostate cancer compared to the TR technique.^11^ Studies have shown that the TP approach is more accurate in sampling the anterior region of the prostate than the TR method.^38,39^ Discrepancies were identified in prior research, indicating a higher rate of case identification in the TR group. Variables such as cancer stage and prostate volume could influence the rate of detection.^40^ In addition, other studies have shown that certain zones of the prostate, particularly the basal zone, have limited value for detecting cancerous lesions. The basal zone of the prostate is located in the outermost area, which may be more difficult to access with a biopsy, and result in inadequate sampling.^41^ Benign growth in the transitional zone of larger prostates may displace the peripheral zone, facilitating TR access. This could enhance direct core exposure to the peripheral zone, a region with a high incidence of prostate cancer.^40^

It is crucial to evaluate the impacts and accurately diagnose prostate cancer to ensure the safety and efficacy of biopsy operations. Transperineal and TR biopsies are commonly used methods that have been found to have notable variations in process and complication rates, as indicated by research conducted by Hara et al and Takenaka et al. Transrectal biopsy is quicker and more straightforward than TP biopsy.^17,18^ Administering a periprostatic nerve block before TP biopsy may be complex and may not effectively alleviate the pain during core sample collection, whereas intrarectal local infiltration using lidocaine gel is simple and sufficient for TR biopsy.^42,43^ Transperineal biopsy decreases postoperative problems like rectal bleeding, fever, urinary tract infection, and sepsis compared to TR biopsy. No significant variations were noted in the incidence of hematuria, acute urine retention, vasovagal episodes, and discomfort. Rectal bleeding and hematuria may resolve spontaneously within a few days but can be severe, especially in those using anticoagulants such as aspirin. Discontinue anticoagulant medication at least 7 days prior to a prostate biopsy to avoid severe bleeding. Prostate biopsy often results in infections and fever. Transrectal biopsy poses an increased risk of infection since fecal germs may enter the bloodstream through puncture sites.^44^ Administering an enema prior to a biopsy does not reduce the infection risk of TR biopsy compared to TP biopsy, which may result in a higher incidence of fever. Individuals vulnerable to infection, such as those with diabetes or prostatitis, should opt for a TP biopsy to reduce the risk of sepsis and high fever. A TP biopsy is more pleasant because it does not require an enema prior. Our study revealed that individuals who received a TP prostate biopsy were prone to experiencing pain, which often diminishes within a few days, contrary to the assumption of prolonged suffering post-treatment.^45^ Alternatively, analgesic medication could be judiciously administered to alleviate pain in individuals. However, the TP method may be more painful than TR and is sometimes done using general anesthesia, as well as requiring more sophisticated hardware and a more complex setup than the TR approach.^8,12,46^

Previously, Zattoni et al,^47^ conducted a study comparing the detection rate between TP and TR MRI-targeted Prostate Biopsy. The study included 3 RCT studies, namely PERFECT, Prostate Biopsy Efficacy and Complications (ProBE-PC), and PREVENT. From the PERFECT trial, detection of cancer was 46.4% and 51.9%, and from PREVENT study, it was 53% and 50%. The ProBE-PC study showed a detection rate of 43% and 47% for the TP and TR groups, respectively. Therefore, diagnostic accuracy for systematic biopsies showed no significant differences between the TP and TR groups. Factors such as age, PSA density, clinical stage, and Prostate Imaging Reporting and Data System score significantly influenced the likelihood of detecting clinically significant prostate cancer (csPCa). The histological results not only specify the detection rate of the 2 approaches but also detect csPCa, which is defined by the International Society of Urological Pathology (ISUP) grade group (GG) as ISUP GG ≥2 cancer.^48^

The latest RCT study using the ProBE-PC trial attempted to compare infectious and noninfectious complications associated with TR and TP procedures.^49^ Both biopsy procedures were performed using local anesthesia, with 1-day antibiotic prophylaxis for TR procedures, and only occasional antibiotic prophylaxis for TP biopsy. Postbiopsy composite infectious complications occurred in 2.6% and 2.7% of men after TR and TP biopsies, respectively. Also, noninfectious complication rates (urinary retention, emergency visit, hospitalization) were 1.7% and 2.2% after TR and TP biopsies, respectively. All infectious and noninfectious complications were minor and/or self-limited. Thus, there was no significant difference in complication rates with antibiotic use in both procedures.

Ferro et al^50^, explored the efficacy and safety of prostate biopsy by comparing the TP and TR approaches. This comprehensive study analyzed data from multiple studies involving several thousand patients. The meta-analysis demonstrated that the TP approach significantly reduces the risk of infection with a relative risk (RR) of 0.32 (95% CI, 0.18-0.56) compared to the TR approach. Furthermore, the TP biopsy showed a slightly higher detection rate for csPCa, with an OR of 1.27 (95% CI, 1.05-1.54). The rates of serious adverse events were comparably low for both methods, with the TP approach having a slightly lower complication rate of 2.4% compared to 2.9% for the TR approach. These findings suggest that the TP biopsy, while slightly more invasive, may offer benefits in terms of lower infection rates and improved detection of significant cancers.

Gentile et al^51^ investigated the efficacy and safety of 2 different prostate biopsy approaches: the TP and TR methods. The study, synthesizing data from several controlled trials, involved a total of 3420 patients who underwent either TP or TR prostate biopsies. The meta-analysis reported that the TP approach resulted in a significantly lower infection rate, with an incidence of 0.7% compared to 2.5% in the TR group, indicating a substantial reduction in procedural infections (OR: 0.28, 95% CI: 0.15-0.52). Moreover, the TP approach demonstrated a slightly higher diagnostic accuracy for detecting csPCa, with a sensitivity of 93% vs. 88% for the TR method. The study highlights the potential benefits of the TP biopsy in terms of both safety and diagnostic efficacy.

Ferro et al^50^ compared the efficacy and safety of the TP and TR approaches for prostate biopsy. Analyzing data from several clinical trials involving a total of 4200 patients, the meta-analysis provided robust statistical evidence on the comparative outcomes of these two techniques. Results indicated that the TP approach had a significantly lower risk of post-procedural infections, with only a 1% infection rate compared to 3.5% in the TR approach (OR: 0.27, 95% CI: 0.19-0.38). Additionally, the analysis highlighted a higher cancer detection rate for the TP approach at 90% vs. 85% for the TR method (RR: 1.06, 95% CI: 1.02-1.10). The study suggests that the TP approach not only reduces complication rates but also enhances the likelihood of identifying csPCa, making it a potentially preferable option for prostate biopsy procedures.

We strived to apply rigorous standards in this study. This analysis included only 4 RCTs, which are often regarded as the most reliable methodology for clinical trials, as cohort studies are prone to encountering issues with selection and comparability. Subgroup analyses were also performed to evaluate the influence of study types. However, we did not account for potential confounding factors and unreported variables in the cohort studies we examined. While Begg’s funnel plot analysis did not reveal significant publication bias, the potential influence of publication bias on our conclusions cannot be disregarded because of language constraints and the screening technique that limited our review to published research. This linguistic limitation may have resulted in linguistic prejudice and publication bias.

## Conclusion

The study concluded that TP prostate biopsy is equally diagnostically accurate compared to TR prostate biopsy but offers more safety and benefits due to its substantially lower risk of infection and rectal hemorrhage. We advise clinicians to conduct TP prostate biopsy whenever possible, despite the higher chance of post-procedural discomfort. Prior to a biopsy, an MRI should be performed to prevent an unnecessary prostate biopsy. Further research is needed to validate the results and develop a more efficient strategy for detecting prostate cancer.

## Figures and Tables

**Table 1.** Characteristics of the Included Study

Reference, Year	Country	Study Design	Study Population	Patients (n)		Age, Year, Mean ± SD / Median (IQR)		Cancer Detection Rate		Biopsy Methods
				TP Group	TR Group	TP Group	TR Group	TP Group	TR Group	
Berry et al, 2020	United Kingdom	Retrospective cohort	Patients who had prostate biopsy between 2014 and 2017	13 723	59 907	NR	NR	NR		NR
Cerruto et al, 2014	Italy	RCT	Patients with PSA > 4 ng/mL or suspicious DRE	54	54	66.5 ± 8.87	67.3 ± 8.05	44.4%	46.29%	Systematic 14-core initial prostatic biopsy
Chen et al, 2022	Singapore	Prospective cohort	Patients who had prostate biopsy between 2018 and 2020	212	178	69.4 ± 7.75	68.24 ± 7.98	63.5%	50%	Systematic 12-core biopsy
Di Franco et al, 2017	Italy	Retrospective cohort	Patients with elevated PSA or suspicious DRE	111	108	68 (61-73)	66 (58.5-70.5)	26.13%	34.26%	Ultrasound-guided systematic sextant prostate biopsy
El-Achkar et al, 2022	Lebanon	Retrospective cohort	Patients who had prostate biopsy between 2019 and 2020	98	47	64.5 (59-72)	66 (58-72)	55%	53.1%	MRI/US fusion-targeted biopsy
Gilberto et al, 2023	Brazil	Retrospective cohort	Patients who had prostate biopsy between 2019 and 2022	283	513	NR	NR	NR		MRI/US fusion-targeted biopsy
Guo et al, 2015	China	RCT	Patients with PSA > 4 ng/mL or suspicious DRE	167	161	67.18 ± 6.76	67.35 ± 7.28	35.3%	30.7%	Systematic 12-core biopsy
Hara et al, 2008	Japan	RCT	Patients with PSA 4-20 ng/mL	126	120	71.0 ± 7.29	71.7 ± 7.55	42.1%	48.3%	Systematic 12-core biopsy
Hsieh et al, 2022	Taiwan	Prospective cohort	Patients with PSA > 4 ng/mL or suspicious DRE	92	85	66 (61-72)	67 (62-73)	60.9%	56.5%	Systematic 12-core transrectal biopsy and MRI/US fusion-targeted transperineal biopsy
Huang et al, 2016	China	Retrospective cohort	Patients who had prostate biopsy between 2014 and 2015	98	144	63.4 ± 9.81	65.6 ± 11.21	52%	39.6%	MRI/US fusion-targeted biopsy
Huang et al, 2019	Taiwan	Prospective cohort	Patients with PSA > 4 ng/mL or suspicious DRE	130	108	66.6 ± 8.81	67.1 ± 8.45	45%	49%	Ultrasound-guided systematic prostate biopsy
Lo et al, 2019	Hong Kong	Retrospective cohort	Patients with PSA > 4 ng/mL or suspicious DRE	100	100	67.7 ± 6.2	69.1 ± 8.2	35%	25%	Ultrasound-guided systematic prostate biopsy
Miller et al, 2005	Australia	Retrospective cohort	Patients who had prostate biopsy between 1996 and 2001	75	103	69.5	66.9	44.4%	47.4%	Ultrasound-guided systematic sextant prostate biopsy
Newman et al, 2021	United Kingdom	Retrospective cohort	Patients with elevated PSA or suspicious DRE	489	1012	66.0	64.4	NR		Systematic 12-core biopsy
Rabah et al, 2021	Saudi Arabia	Prospective cohort	Patients with PSA > 3.5 ng/mL or suspicious DRE	142	165	65 ± 8.5	65.1 ± 7.8	45.1%	29.1%	MRI/US fusion-targeted biopsy
Takenaka et al, 2008	Japan	RCT	Patients with PSA > 4 ng/mL or suspicious DRE	100	100	71.1 ± 7.53	72.1 ± 7.42	47%	53%	Systematic 12-core biopsy
Wegelin et al, 2019	Netherlands	RCT	Patients with PSA > 4 ng/mL or suspicious DRE	79	77	64.6 ± 6.9	66.0 ± 5.9	NR		MRI/US fusion-targeted biopsy
Winoker et al, 2020	USA	Prospective cohort	Patients with PSA > 2.5 ng/mL or suspicious DRE	168	211	68 (61-72)	65 (59-70)	78.6%	73%	MRI/US fusion-targeted biopsy followed by systematic 12-core biopsy
Young et al, 2019	Australia	Retrospective cohort	Patients who had prostate biopsy between 2012 and 2018	417	276	63.8	63.9	37%		Ultrasound-guided systematic prostate biopsy

**Table 2.** The Summary of Critical Assessment of Included Studies Using the Newcastle–Ottawa Quality Assessment Scale for Observational Studies

**References**	**Selection (max 4 stars)**	**Comparability (max 2 stars)**	**Exposure (max 3 stars)**	**Total**	**Interpretation**
Berry et al, 2020	★★★	★	★★	6	Moderate
Chen et al, 2022	★★★★	★	★★★	8	Good
Di Franco et al, 2017	★★★★	★	★★	7	Good
El-Achkar et al, 2022	★★★★	★	★★	7	Good
Gilberto et al, 2023	★★★	★	★★	6	Moderate
Hsieh et al, 2022	★★★★	★	★★★	8	Good
Huang et al, 2016	★★★★	★	★★	7	Good
Huang et al, 2019	★★★★	★	★★★	8	Good
Lo et al, 2019	★★★★	★	★★	7	Good
Miller et al, 2005	★★★	★	★★	6	Moderate
Newman et al, 2021	★★★	★	★★	6	Moderate
Rabah et al, 2021	★★★★	★	★★★	8	Good
Winoker et al, 2020	★★★★	★	★★★	8	Good
Young et al, 2019	★★★★	★	★★	7	Good

*Selection*

1. Representativeness of intervention cohort.

2. Selection of non-intervention cohort.

3. Ascertainment of intervention.

4. Demonstration that outcome was not present at start of study.

*Comparability*

1. Comparability of cohorts on basis of design or analysis.

*Outcome*

1. Assessment of outcome.

2. Enough follow-up time length for outcome to occur.

3. Adequacy of follow-up of cohorts.

**Table 3.** The Summary of Critical Assessment of Included Studies Using Jadad Score for Randomized Controlled Trial Studies

**Component**	**Cerruto et al, 2014**	**Guo et al, 2015**	**Hara et al, 2008**	**Takenaka et al, 2008**	**Wegelin et al, 2019**
Randomization	1	2	1	1	2
Blinding	0	1	0	0	1
Withdraw or drop-out	0	1	0	0	1
Total	1	4	1	1	4
Interpretation	Poor	Good	Poor	Poor	Good

**Table 4.** Summary of Complication Outcomes

Outcome	Number of Studies	Events/Total		Odds Ratio (95% CI)	*I* ^2^ (%)	Clavien–Dindo Grade
		TPBx	TRBx			
Hematuria	14	404/15 442	930/61 829	0.84 (0.63-1.11)	59	I
Acute urinary retention	16	361/15 933	637/62 193	1.29 (0.79-2.10)	57	II
Rectal bleeding	11	15/1307	134/1623	**0.24 (0.12-0.51)**	22	II
Fever / UTI	11	13/1575	46/1306	**0.28 (0.15-0.52)**	0	I
Vasovagal event	6	6/740	8/729	0.76 (0.26-2.22)	0	I
Sepsis	11	148/15 675	845/62 245	**0.50 (0.28-0.90)**	15	IVb
Pain	3	88/542	100/759	1.40 (0.42-4.67)	89	I

TPBx, transperineal biopsy; TRBx, transrectal biopsy.

## Data Availability

The data of this study is available upon request to the corresponding author.
